# Eggshell waste derived nano-hydroxyapatite/metakaolin composites for bone scaffold applications

**DOI:** 10.1039/d6ra01169a

**Published:** 2026-04-17

**Authors:** Zaid Kareem, Ersan Eyiler

**Affiliations:** a Prosthetics and Orthotics Engineering Department, University of Kerbala 1152 Karbala Iraq zaltaey88@gmail.com +964-772-377-9470; b Advanced Materials and Nanotechnology Department, Cukurova University 01250 Adana Turkey; c Department of Chemical Engineering, Cukurova University 01250 Adana Turkey; d Tissue Engineering Department, Cukurova University 01250 Adana Turkey

## Abstract

This study presents a novel, zero-waste wet chemical precipitation method for synthesizing eggshell-derived nano-hydroxyapatite (HA) reinforced with metakaolin (MK) for potential biomedical applications. Calcium oxide obtained from calcined eggshells served as the calcium source, and MK was incorporated at varying weight ratios (25–100% of HA mass) prior to cold pressing. The composites were characterized using XRD, FTIR, SEM-EDX, and BET analyses to assess phase composition, functional groups, microstructure, and surface area. Results confirmed successful HA formation with rod-like nanocrystals, while increasing MK content reduced crystallinity and crystallite size (37.3–21.1 nm) due to aluminosilicate incorporation. Cold pressing decreased porosity (63–55%), increased bulk density (1.57–1.66 g cm^−3^), and improved mechanical performance, with compressive strength and diametral tensile strength reaching 16.4 MPa and 11.8 MPa, respectively, within the range of cancellous bone. *In vitro* bioactivity tests revealed progressive apatite layer formation over 21 days, while antimicrobial assays showed broad-spectrum inhibition against Gram-positive, Gram-negative, and fungal strains. MTT cytotoxicity assays using MC3T3-E1 pre-osteoblasts demonstrated high cell viability (>95%) at ≤12.5 µg mL^−1^, indicating good biocompatibility. The developed HA/MK composites exhibited promising mechanical, bioactive, and antimicrobial properties, supporting their potential use in bone tissue engineering scaffolds.

## Abstract 2

This study presents a novel, zero-waste wet chemical precipitation method for synthesizing eggshell-derived nano-hydroxyapatite (HA) reinforced with metakaolin (MK) for potential biomedical applications.

## Graphical abstract

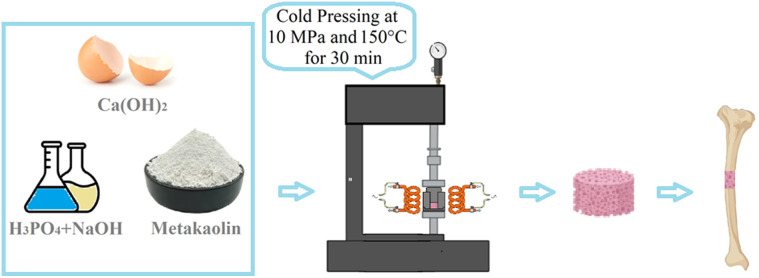

## 1. Introduction

Eggshells are biowaste materials, constituted, structurally speaking, by a three-layered structure, namely the cuticle, the spongeous layer and the lamellar layer.^1^ The cuticle layer represents the outermost surface, and it consists of several proteins, spongeous and lamellar layers form a matrix constituted by protein fibers bonded to calcite (calcium carbonate) crystals in the proportion of 1 : 50.^2^ The eggshell represents 11% of the total weight of the egg and is composed by calcium carbonate 94%, calcium phosphate 1%, organic matter 4% and magnesium carbonate 1%.^3^

According to FAO, in 2023, world egg production reached 97 million tons in an increasingly trend from the previous year.^4^ Consequently, the management of large amounts of eggshell waste annually produced in the world is problematic as generally this material is only disposed at landfills with odor production and microbial growth.^5^ The utilization of bio-calcium found in eggshells in various applications contributes to reducing the negative impacts of untreated eggshell waste, as well as saving disposal costs in landfills.

Commercially available HAs are expensive due to the use of high purity reagents. On the other hand, HAs derived from natural materials such as bovine bone, fish bone or coral have the advantage that they inherit some properties of the raw materials such as the pore structure, carbonated HA *etc.*^3^ In biomaterials fields, eggshells are exploited in HA (Ca_10_(PO_4_)_6_(OH)_2_) synthesis *via* wet chemical precipitation. Many researchers have extensively studied the synthesis of HA based on eggshells *via* wet chemical precipitation in last decade,^6–8^ and they focused on advancing and optimizing the process of producing HA from eggshells.

Wet chemical precipitation method is common method for nanoscale hydroxyapatite synthesis, and many researchers followed this method in producing HA based eggshells. The wet chemical precipitation method permits chemists to control morphologies, particle size, and incorporating ions into the chemical structure of HA, however all these options aimed to enhance the characteristics of HA. Despite the many positive features of this method, it is still having a limitation presenting in the low yielding (limited for laboratory-scale). In conventional wet chemical precipitation methods for hydroxyapatite synthesis, the overall material yield is often reduced due to multiple washing and filtration steps required to remove residual ions from the reaction medium. Hydroxyapatite particles are typically nanosized and highly dispersed, a portion of the precipitated material may remain suspended in the supernatant and be lost during washing or filtration processes. In addition, the incomplete precipitation and the formation of transient calcium phosphate phases may further reduce the effective recovery of the final HA product. In the present approach, the elimination of conventional washing and filtration steps minimizes the loss of fine particles and enables nearly complete recovery of the precipitated material, thereby improving overall process efficiency.

Literately, several researchers have explored methods to scale up the production of HA from eggshell biowaste, aiming to improve yield and process efficiency. For instance, Muthu *et al.* optimized HA synthesis using a microwave-assisted wet chemical precipitation method at both laboratory and pilot scales, achieving a 45% conversion of eggshell biowaste into HA with enhanced process speed and energy efficiency.^9^ Similarly, Agbeboh *et al.* compared two wet precipitation approaches, revealing that the orthophosphoric acid route produced HA with higher purity, whereas the nitric acid-based method offered finer particle size, improved safety, faster reaction time, and greater environmental sustainability.^10^ These studies highlight innovative approaches to increasing yield, improving particle characteristics, and ensuring eco-friendly production, which are critical steps toward large-scale application of eggshell-derived HA. Another major challenge in utilizing hydroxyapatite for scaffold fabrication lies in its inherent mechanical limitations, despite its excellent bioactivity. Pure HA is brittle and exhibits low fracture toughness, which restricts its application in load-bearing biomedical implants. To address this issue, recent research has focused on reinforcing HA with complementary materials to enhance its physical and mechanical properties without compromising its biocompatibility. In literature, hydroxyapatite has been widely reinforced with various organic and inorganic materials to enhance its mechanical, thermal, and biological properties. For instance, natural polymers such as chitosan have been incorporated into HA matrices to improve biocompatibility, antibacterial properties, and biodegradability.^11,12^ Similarly, synthetic polymers like polycaprolactone (PCL) have been used to enhance flexibility and processability, particularly in scaffold fabrication for tissue engineering applications.^13^ Polylactic acid (PLA) has also been employed as a reinforcing agent due to its excellent biodegradability and suitability for 3D printing and biomedical implants.^14,15^ In addition to polymeric reinforcements, inorganic materials such as silica have been introduced to HA to improve its mechanical strength, chemical stability, and osteoconductive behavior.^11^ Furthermore, kaolin, a naturally occurring aluminosilicate clay, has been reported as a cost-effective reinforcement for HA-based composites, offering improved compressive strength and structural stability.^16–18^ These reinforcements collectively aim to overcome the inherent brittleness and low fracture toughness of pure HA, thereby expanding its potential applications in orthopedic, dental, and tissue engineering fields.

This study aimed to synthesize hydroxyapatite from eggshells using a modified wet chemical precipitation method that ensured zero material loss, as it eliminates conventional waste-generating steps such as washing and filtration for the precipitated nano-HA. In addition, the developed process was utilized to produce HA reinforced with calcined kaolin (the common term is metakaolin), creating a composite material suitable for cold pressing and molding. This approach not only maximizes material yield and sustainability but also enhances the mechanical properties of HA, making the composite promising for potential biomedical applications.

## 2. Materials and method

### 2.1 Materials

The eggshells were collected from domestic waste. *Ortho*-phosphoric acid solution (85.5% purity) and sodium hydroxide pellets (EMSURE®, assay ≥99%) were supplied from Sigma Aldrich. Calcined kaolin or metakaolin (MK) (KAOCAL ® 80 Calcined kaolin) was delivered from AVS mineral company, Türkiye.

### 2.2 Preparation of calcined kaolin reinforced hydroxyapatite composites from eggshells

Eggshells were collected, cleaned with tap water and brush, followed by a water-boiling process for 1 h. Then they were dried in oven for 2 h at 150 °C. Grinding process was applied to the dried eggshells for 5 min using ordinary grinding machine (200 g capacity and 1000 W power) to obtain eggshell powder. Next, it was transferred to muffle furnace for decarbonization by heat treatment at 900 °C for 3 h. The resultant ash was soaked in distilled water and stirred (magnetically) for the hydrating process, then the water was spelt out, and the rest powder was dried at 100 °C overnight. The calcined eggshell powder (CES) was grinded again for 5 min and sieved using 75 µm sieve.

**Fig. 1:**
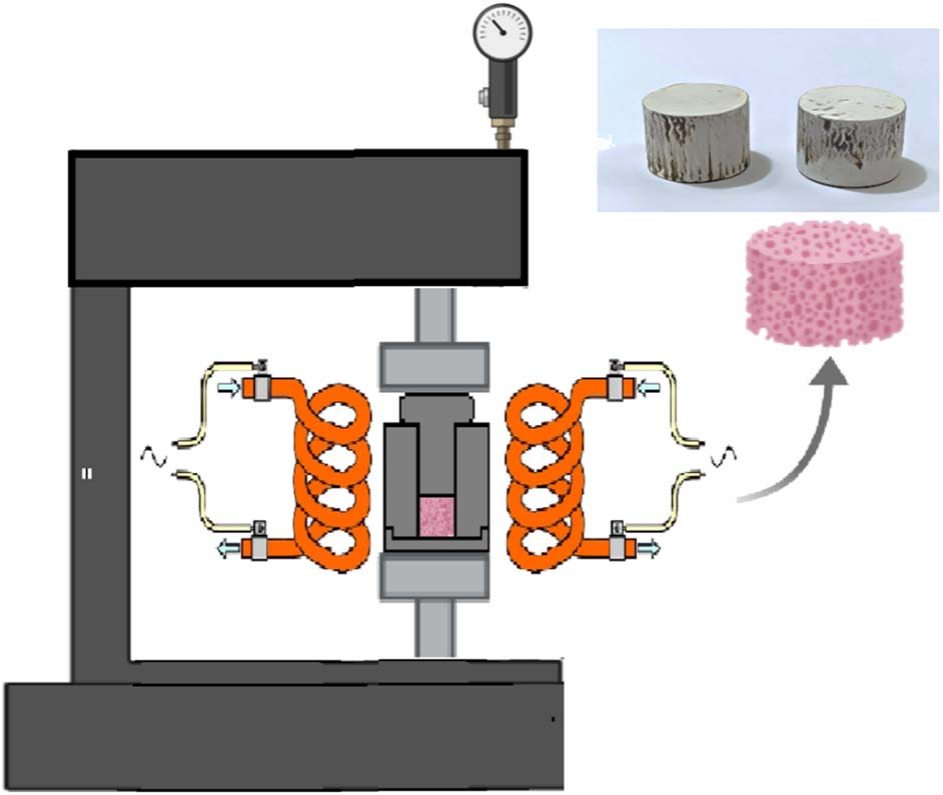
Cold pressing apparatus.

Nanoscale hydroxyapatite was synthesized using eggshell-derived calcium as the primary calcium source. 0.4 L of H_3_PO_4_ solution of 0.67 M was added (10 drops per minute) under magnetic stirring (fixed at 700 rpm) to solution of 1 M of Ca(OH)_2_, prepared by dissolving 29.6 g in 0.4 L distilled water, due to the limited solubility of Ca(OH)_2_, the system represents a suspension rather than a completely dissolved solution. 0.05 L of NaOH solution of 1 M was used as a buffer solution to adjust pH of the solution. After the dropwise addition of the H_3_PO_4_ solution was completed (about 6 h), the mixture was stirred continuously for an additional 6 h, and then the suspension was left to precipitate overnight. From the experiment, it was concluded that the selected conditions and concentrations yielded 45 g of HA. Subsequently, MK was added to the HA suspension in four proportions (25%, 50%, 75%, and 100% of the synthesized HA mass (*i.e.*, 45 g)) and stirred for 6 h. Then, the suspension was poured into shallow dish to increase surface area and dried under vacuum at 60 °C and 10 mbar. Then, the powder was dried again in oven overnight at 100 °C. The oven dried powder (OD) was grinded again for 1 min and mixed with water at 10% by mass of OD. The wet mixture was put into stainless steel mold for CP under 10 MPa, 150 °C and 30 min (holding time) (Fig. 1). A mold with a diameter of 1.2 cm was used, and the height of each sample was controlled by adjusting the weight of each batch prepared for cold pressing process. Cylindrical samples of 1.2 cm in diameter and 2.4 cm in height were produced for compressive strength test, while cylindrical discs of 1.2 cm in diameter and 0.6 cm in height were used for other mechanical, physical and biological tests.

### 2.3 Characterization

#### 2.3.1 X-ray diffraction

The phase purity of HA and MK and composite powders was determined using an X-ray diffractometer (Rigaku Ultima IV) operated at 40 kV and 30 mA utilizing CuKα radiation, at a step size of 0.02° and a step time of 0.5 second. All diffractograms were obtained in the range of 5–60° of 2*θ* angles. Phase identification was carried out by comparing the diffraction data with standards of Joint Committee on Powder Diffraction Standards (JCPDS).

#### 2.3.2 SEM-EDX

To examine the surface morphology and determine the Ca/P ratio of the sample, Field Emission Scanning Electron Microscopy (FESEM) equipped with Energy Dispersive X-ray Spectroscopy (EDX) using a MIRA3 TESCAN instrument was performed at various magnifications. The samples were coated with a thin layer of gold (Au) prior to FESEM-EDX analysis to enhance surface conductivity and improve imaging quality.

#### 2.3.3 Fourier transform infrared spectroscopy

Fourier Transform Infrared (FTIR) spectroscopy was conducted using an FTIR-8300 spectrometer (Shimadzu, Japan) to identify functional groups present in the samples. The spectra were recorded over a frequency range of 4000–500 cm^−1^.

#### 2.3.4 BET

The surface area, pore diameter and total pore volume of the samples were determined *via* conducting BET analysis (Micromeritics, USA).

#### 2.3.5 Mechanical and physical properties of the composites

The mechanical properties of the samples were evaluated by measuring their compressive strength and Young's modulus. Compression tests were performed using a universal testing machine equipped with a 5-ton load cell, operated at a constant crosshead speed of 2.00 mm min^−1^. For each sample, the average value of three specimens was calculated.

The Vickers hardness of HA/MK composites was measured in accordance with ASTM E384 using a Vickers microhardness tester (HMV-2, Shimadzu, Japan) with a specified load and dwell time. Hardness values were calculated using:

1 VHN = 1.854(*P*/*d*^2^)

where *P* is the applied load in newtons (N) and *d* is the average indentation diagonal in millimeters (mm).

Diametral tensile strength (DTS) was determined using a universal testing machine (Instron 3369, Instron Corp., USA) at a crosshead speed of 1 mm min^−1^. DTS values were calculated from:

2 DTS = 2*P*/π*Dt*

where *P* is the fracture load in newtons (N), *D* is the specimen diameter (mm), and *t* is its thickness (mm).

Porosity percentage was calculated from the eqn (3):3
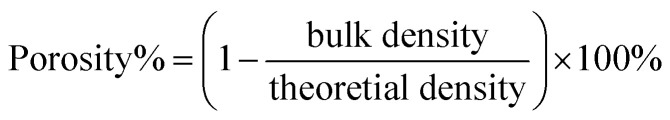


Theoretical density of the composite was calculated using the rule of mixtures, which considers the contribution of each constituent based on its weight fraction. The eqn (4) was applied:4
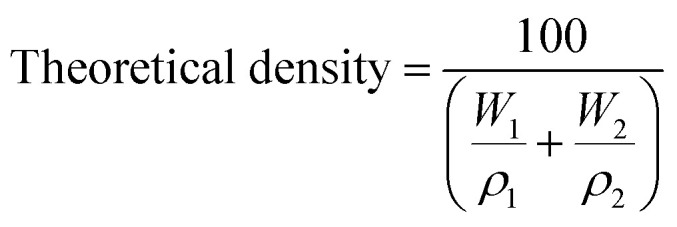
where *W*_1_ and *W*_2_, are the weight percentages of MK and HA, respectively. *ρ*_1_ and *ρ*_2_, are their corresponding densities (in g cm^−3^).

The densities used in the calculation were 2.50 g cm^−3^ for MK, 3.16 g cm^−3^ for HA. Bulk density was determined using Archimedes' principle by measuring the dry and submerged weights of the samples with a density tester. The values were then used in the eqn (5):5
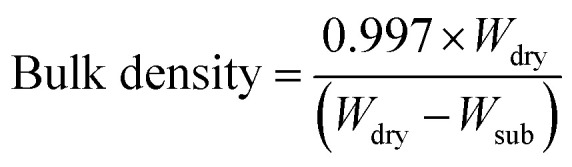


Biodegradability studies were conducted to assess the gradual weight loss of the samples in a phosphate buffer solution (PBS, pH 7.4) for 7, 14 and 21 days. Each pellet was immersed in 10 mL of PBS solution and kept at RT in closed container. Readings were taken for the pH variation and weight loss of each respective pellet before and after the incubation using a pH meter and an analytical balance (accuracy: 0.001 g). The samples were then dried in oven at 100 °C for 3 h before weight recording. The percentage of weight decrease (*W*_d_) was calculated using the eqn (6):6
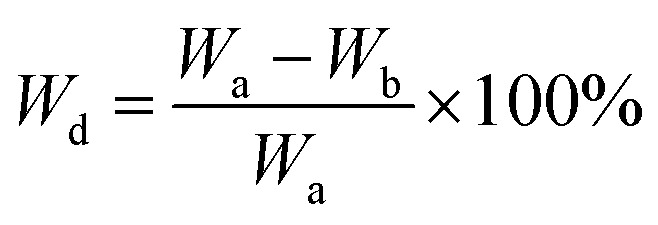
where *W*_a_ and *W*_b_ are the respective weights of samples before and after soaking in phosphate buffer saline solution, respectively.

#### 2.3.6 Biocompatibility and biofunctionality

The bioactivity of the prepared samples was investigated using a simulated body fluid (SBF) solution, which was prepared according to the protocol of.^19^ The pellets representing the synthesized samples were placed in an air-tight polyethylene container with a volume of 50 mL SBF solution for a period of 7, 14 and 21 days, respectively at 37 °C. SEM-EDX analysis was conducted to find out the precipitation of apatite on the surface of samples. The variations in the EDX spectra were considered as an indication of compositional changes associated with apatite formation.

Cytotoxicity was assessed *via* MTT assay on MC3T3-E1 pre-osteoblastic mouse cells; MC3T3-E1 cells were obtained from the Iranian Biological Resource Center (IBRC). Cells were cultured in α-MEM with 10% FBS and 1% penicillin/streptomycin at 37 °C, 5% CO_2_. They were seeded (5000–10 000 cells per well) in 96-well plates and incubated for 24 h. Serial dilutions of the material were applied for 48 h, followed by MTT addition (5 mg mL^−1^). Formazan was dissolved in DMSO, and absorbance was read at 570 nm to determine cell viability (%). The antimicrobial activity of the samples was evaluated using the agar well diffusion method against *Staphylococcus aureus*, *Staphylococcus epidermidis* (Gram-positive), *Escherichia coli*, *Pseudomonas aeruginosa* (Gram-negative), and *Candida albicans* (fungal model). Microbial suspensions were prepared from fresh cultures, standardized to a 0.5 McFarland turbidity standard (1.5 × 10^8^ CFU mL^−1^) for reproducibility, and inoculated (0.1 mL) onto Mueller–Hinton agar plates to achieve uniform lawn growth. After 10–15 min equilibration, four 5 mm wells were aseptically made per plate, and 50 µL of the sterile-prepared samples at concentrations of 1000, 500, 250, and 125 µg mL^−1^ were loaded into the wells. Plates were incubated at 37 °C for 18–24 h for bacterial strains and up to 48 h for *C. albicans*. Inhibition zones were measured in millimeters using a digital caliper to quantify antimicrobial activity.

### 2.4 ANOVA statistical analysis

The compressive strength, DTS, hardness and Young's modulus were performed in triplicate. Data are presented as mean ± standard deviation. Statistical significance between groups was assessed using one-way ANOVA using Minitab software (Minitab® 22.1). A *p*-value ≤0.05 was considered statistically significant.

## 3. Results and discussion

### 3.1 X-ray diffraction

**Fig. 2:**
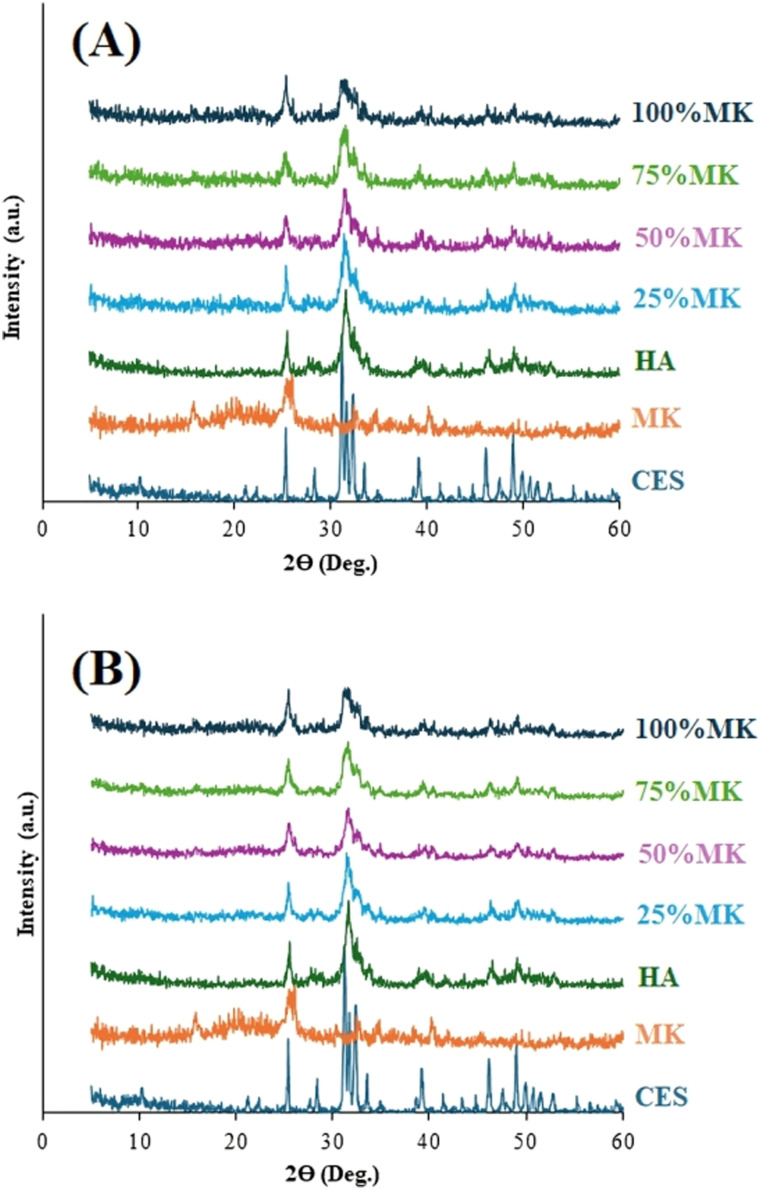
XRD patterns of the precursors and HA/MK composites: (A) OD samples and (B) CP samples.

X-ray diffraction patterns for both oven dried (OD) and (CP) HA/MK composites with varying MK contents (25%, 50%, 75%, and 100%) were analyzed in comparison with reference reacted precursor; calcined eggshell powder (CES), MK, and HA (Fig. 2). The XRD pattern of CES exhibited characteristic calcite peaks primarily at 2*θ* = 29.4°, 39.5°, and 47°, confirming the crystalline nature of the CES.^20–22^ The HA showed sharp, well-defined characteristic peaks at approximately 2*θ* = 25.9°, 31.8°, 32.9°, 34.0°, and 49.5°, corresponding to planes (002), (211), (300), (202), and (213), respectively, matching the standard hexagonal hydroxyapatite (JCPDS 09-0432). The XRD pattern of pure MK demonstrated a broad amorphous halo between 15° and 35°, consistent with its disordered or amorphous aluminosilicate structure following calcination.^23–25^ Other estimated phases are amorphous zeolitic phases, which appears as a broad, diffuse peak or halo instead of the sharp, distinct peaks characteristic of a crystalline material.^26,27^ All composite samples showed characteristics, indicating the integrity of HA and confirming the successful HA formation regardless of MK incorporation levels. However, an increase in MK content led to decreased peak intensity and increased peak broadening in both before and after cold pressing, indicating reduced crystallinity. However, this reduction in crystal growth may result from structural disorder or inhibited crystal growth due to the presence of amorphous MK phases or the incorporation of MK oxides into the chemical structure of HA. Many papers have reported that doping process led to a reduction in HA crystallinity.^28,29^ Studies on Si- and Al-doped HA have indicated that Al tends to substitute for Ca in the HA lattice, whereas Si partially replaces phosphate (PO4)^3−^ groups upon incorporation.^30–32^ However, Si doping improved the biodegradability, resorbability and mineralization ability of HA, which can enhance bone scaffold performance by facilitating faster apatite formation and promoting improved bone tissue integration.^31,33^ Meanwhile, Al enhanced its thermal stability, contributing to improved structural stability of the bone scaffold.^34^

The crystallite size of the HA phase was estimated using the Scherrer equation, based on the prominent diffraction peak at 2*θ* ≈ 31.8°, attributed to the (211) plane:

7 *D* = *Kλ*/*β* cos *θ*

where *D* is the crystallite size (nm), *K* is the shape factor (0.9), *λ* is the Cu Kα wavelength (0.15418 nm), *β* is the full width at half-maximum (FWHM) in radians, and *θ* is the Bragg diffraction angle.

The calculated crystallite sizes are presented in Table 1.

**Table 1:** Crystallite sizes as a function to MK%

Sample composition	Crystallite size (nm) of OD	Crystallite size (nm) of CP
25% MK	36.7	37.3
50% MK	31.1	30.8
75% MK	25.3	26.0
100% MK	21.3	21.1

The crystallite size decreased significantly from approximately 36.7 nm and 37.3 nm for the 25% MK composite of OD and CP, respectively, to about 21.3 nm and 21.1 nm for the 100% MK composite of OD and CP, respectively, and this decrease in crystallite size confirms that increased MK content significantly impacts HA crystal growth, resulting in reduced crystallinity and enhanced structural disorder. The presence of the amorphous aluminosilicate phase (MK) appears to disrupt crystallization processes, causing nucleation and growth constraints. In the comparison between OD and CP samples, cold pressing had no significant effect on the crystallite size of HA. This outcome may be attributed to the low processing temperature, which was insufficient to induce any noticeable changes in the HA structure.

### 3.2 Fourier transform infrared spectroscopy

**Fig. 3:**
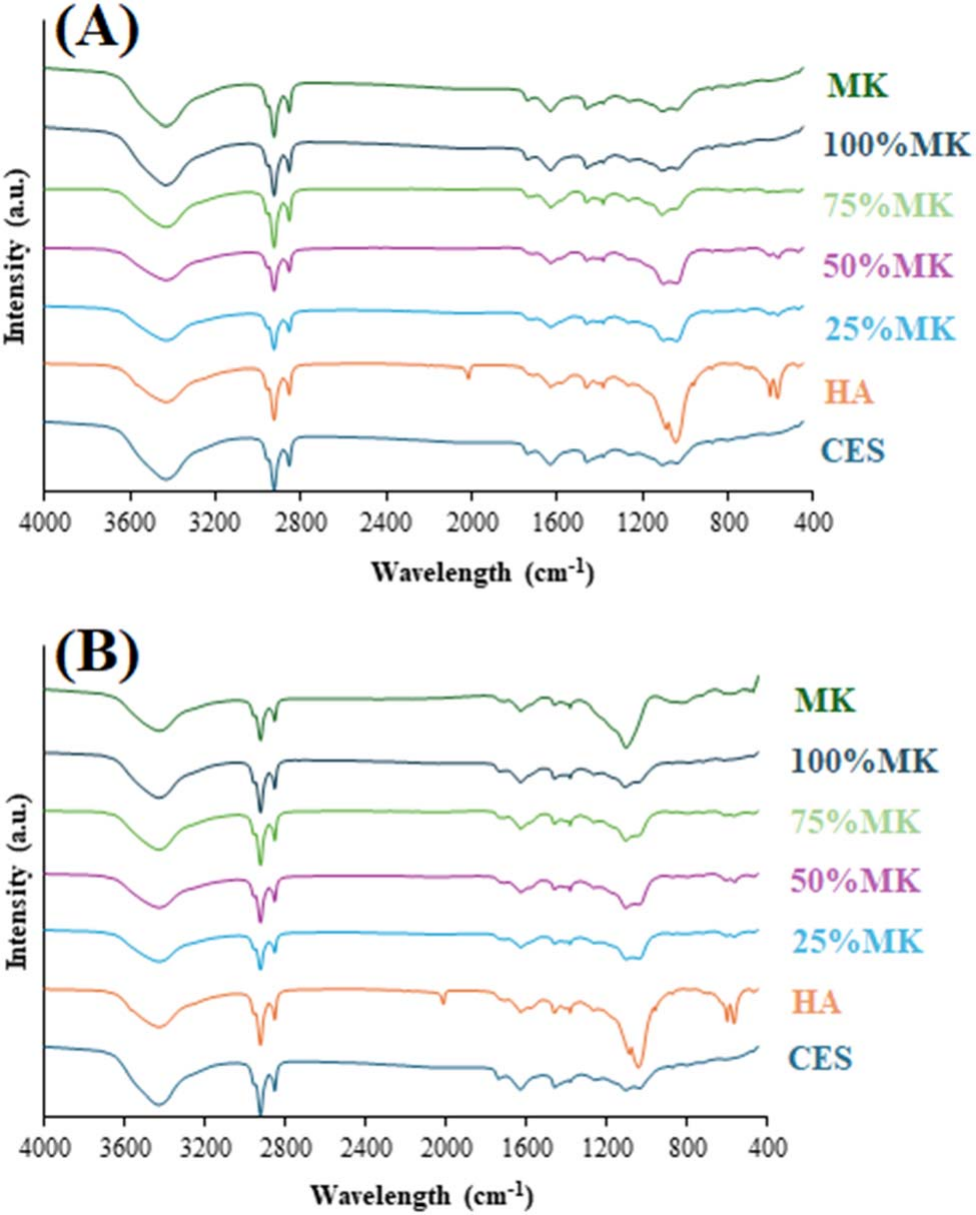
FTIR spectra of the precursors and HA/MK composites: (A) OD samples and (B) CP samples.

FTIR spectroscopy was employed to HA/MK composites of both OD and CP, HA, CES, and MK (Fig. 3). The FTIR spectrum of HA exhibits characteristic phosphate (PO_4_^3−^) vibrational bands prominently at approximately 560–610 cm^−1^ (bending mode, *ν*_4_),^35,36^ 960 cm^−1^ (symmetrical stretching mode, *ν*_1_),^36^ and between 1000–1100 cm^−1^ (asymmetrical stretching mode, *ν*_3_).^35,36^ Additionally, a clear hydroxyl (OH^−^) absorption peak near 3570 cm^−1^ confirms the presence of structural hydroxyl groups characteristic of stoichiometric HA.^10,22^ The calcined eggshell spectrum shows distinctive carbonate bands, primarily observed at approximately 1420–1480 cm^−1^, corresponding to the carbonate group (CO_3_^2−^),^22,35^ and confirming residual calcium carbonate phases. The absorption peak appears at 3450 to 3600 cm^−1^, indicating to O–H stretching vibrations that confirm the formation of Ca(OH)_2_.^36,37^ The MK spectrum shows characteristic Si–O–Si and Al–O–Si vibrational bands in the range of 1000–1200 cm^−1^,^38,39^ and O–H stretching vibrations appear as broad absorption peaks around 3450–3600 cm^−1^,^23,40^ indicating hydroxyl groups bonded to the aluminosilicate structure. For the composite samples (25%, 50%, 75%, and 100% MK), the FTIR spectra reveals a combination of HA and MK characteristics. At lower MK contents (25–50%), prominent HA bands at around 560–610 cm^−1^, 960 cm^−1^, and 1000–1100 cm^−1^ remain well-defined, indicating the preservation of crystalline HA structure. The carbonate band at 1420–1480 cm^−1^ is still present, but significantly reduced, suggesting minor carbonate substitution within the HA lattice or incomplete carbonate removal. As MK content increases (75–100%), phosphate bands become noticeably broader and reduced in intensity, indicative of reduced crystallinity and increased amorphous character, which aligns well with XRD results. Furthermore, the intensity of the broad Si–O–Si/Al–O–Si vibration between 1000–1200 cm^−1^ becomes more prominent, indicating increased MK presence and contribution. The broadening in the O–H absorption between 3400–3600 cm^−1^ suggests increased structural disorder and hydration interactions, likely related to aluminosilicate interactions or adsorbed moisture. Overall, FTIR results corroborate the XRD analysis, confirming that HA crystallinity decreases with higher MK incorporation, leading to increased structural disorder and a more amorphous composite structure. The presence of aluminosilicate-related vibrations demonstrates chemical integration between Ca with Si or Al. Previous researchers confirmed the Ca replacement with Al or Si when HA was doped with silica and alumina oxides.^30,34,41^ Al or Si doped HA was primarily characterized by enhanced physicochemical, structural, and biological properties, making it superior to pure HA for biomedical applications.^31,32^

### 3.3 SEM-EDX

**Fig. 4:**
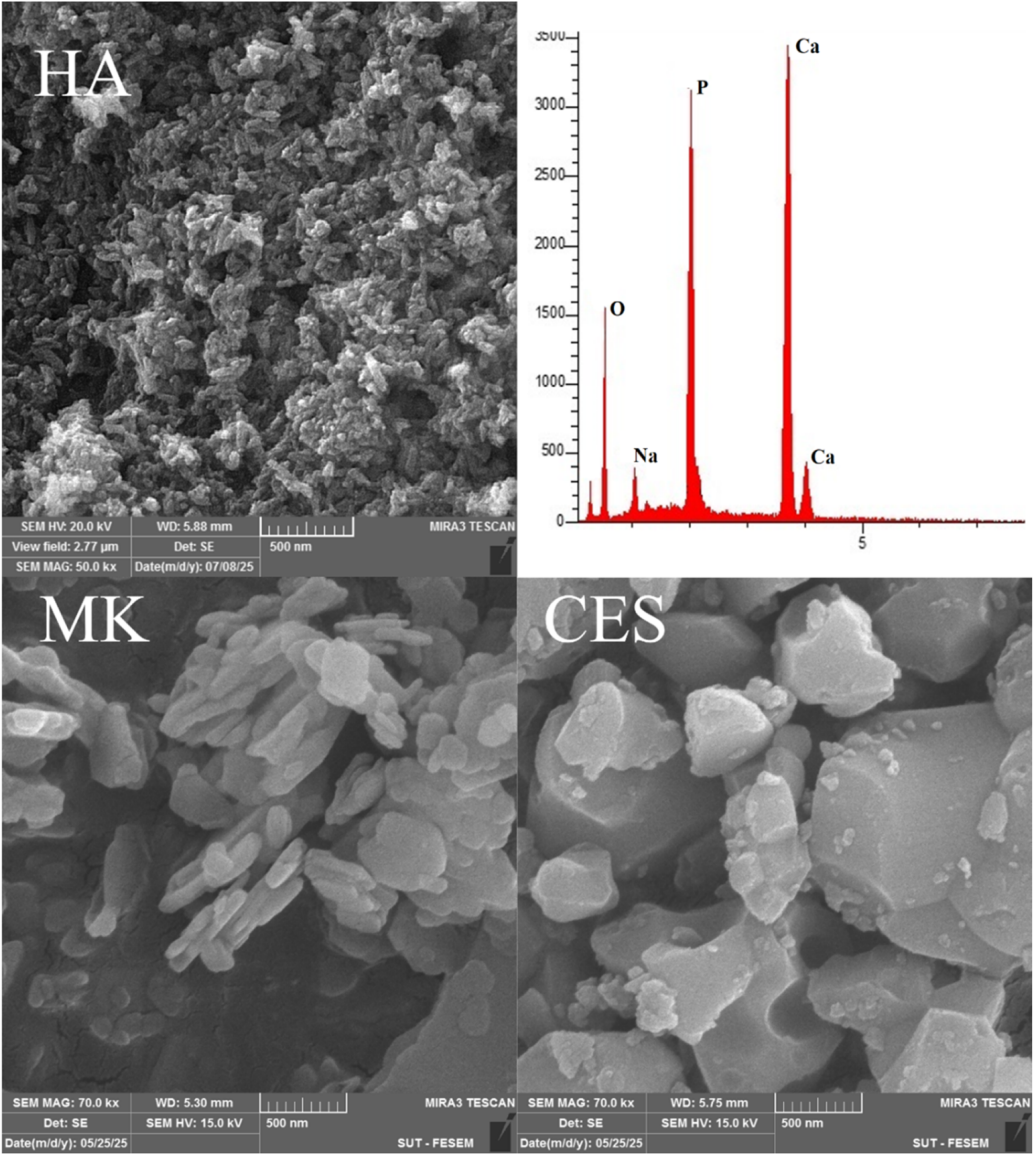
SEM-EDX analysis of HA and SEM micrographs of MK and CES powder.

The microstructures of HA, MK and CES are shown in Fig. 4. The image revealed the formation of nanoscale HA particles with rod-like morphology. It was reported that rods like HA was synthesized successfully from eggshell.^42,43^ The approximated dimensions of nano-rod hydroxyapatite ranged between 150-200 nm in length and 30–50 nm in width. The pores area clearly appeared in 200 nm magnifications indicative of a large surface area.^44^

**Fig. 5:**
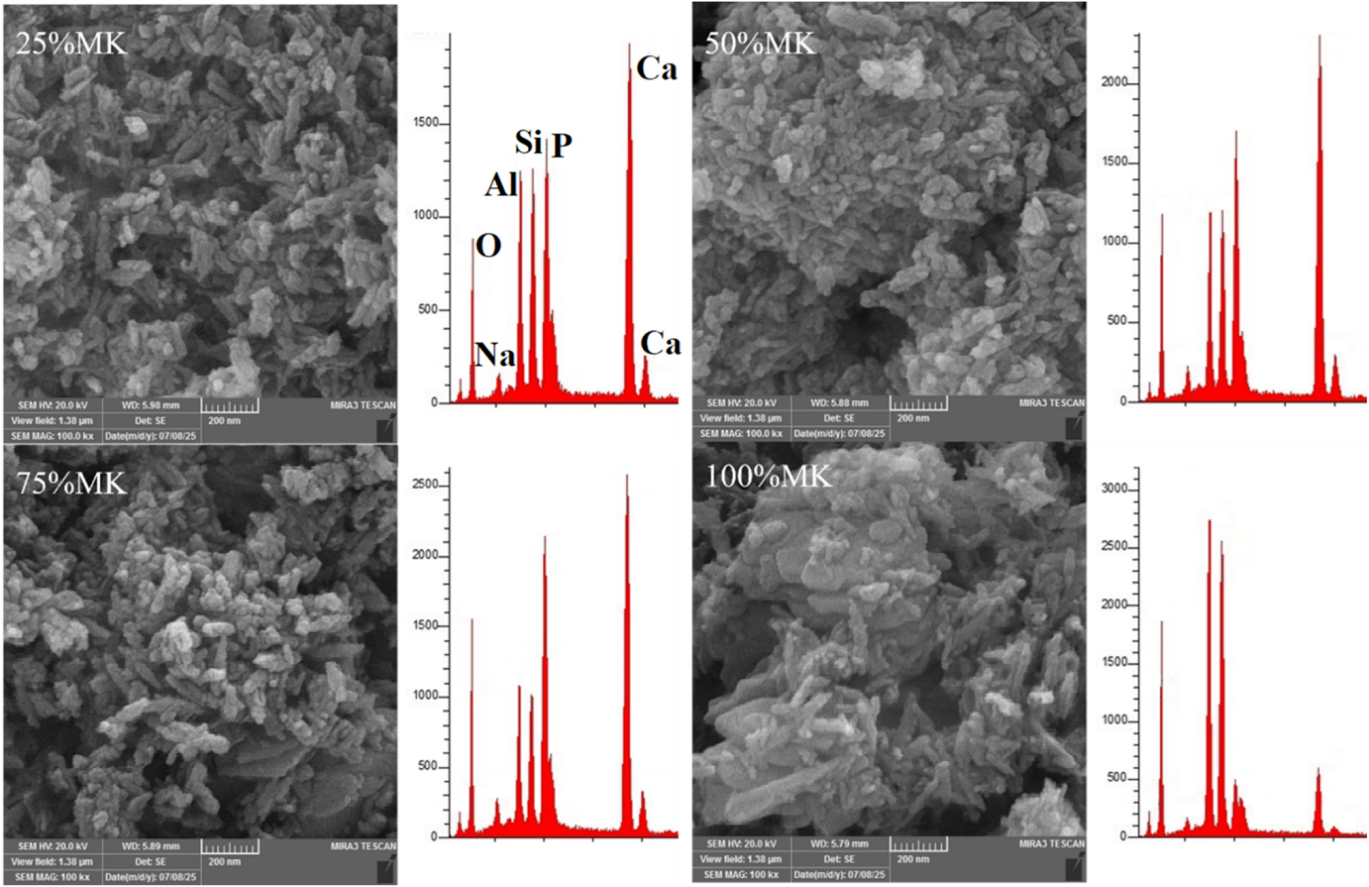
SEM-EDX analysis of HA/MK composites with varying MK contents (25%, 50%, 75%, and 100%)-OD.

**Fig. 6:**
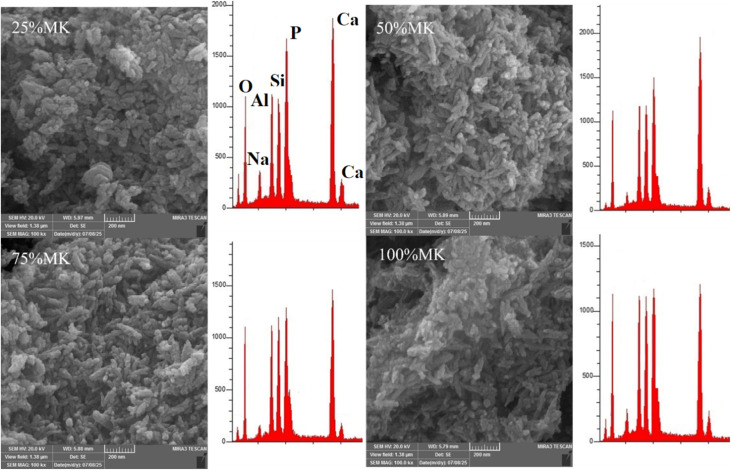
SEM-EDX analysis of HA/MK composites with varying MK contents (25%, 50%, 75%, and 100%)-CP.

The microstructures of MK/HA composites (OD and CP) for the four percentages (25%, 50%, 75%, and 100%) are present in Fig. 5 and 6. The images confirmed the stability of rod like HA in the four percentages even after CP. Metakaolin appeared as a microscale plate particles covered by the nanoparticles of HA. The pores between the metakaolin particles looked big in size and devour HA nanoparticles, meanwhile the pore got narrower for counterpart sample after CP, see Fig. 6. In comparison with MK microstructure shown in Fig. 4, there was disintegration in MK particle size into smaller size, and it was confirmed that the MK was disintegrated under alkaline conditions.^45^ The CES particles, which appeared as polygonal shapes, were clearly observed in the HA and HA/MK microstructure images, confirming that all CES was consumed during the HA synthesis process. Signs of zeolite grain initiation appeared as bright clusters of particles.

The EDX results of HA and HA/MK composites are shown in Fig. 4–6. Regarded to HA Ca/P atomic ratio was 1.86 indicating to Ca rich HA.^46^ The sodium of buffered solution appeared in HA EDX result. Upon the incorporation of metakaolin, new peaks corresponding to (Al) and (Si) appeared. As the MK content increased from 25% to 100%, the intensity of the Al and Si peaks progressively increased, while the Ca and P peaks decreased, reflecting the gradual replacement of the HA phase by MK or MK oxides incorporation in crystal structure of HA. It has been reported that Si was replaced by phosphate,^47^ while Al was replaced by Ca.^48,49^ At higher MK concentrations, especially at 75% and 100%, the spectra were dominated by aluminosilicate components, these observations demonstrated the effective integration of MK into the HA structure and the compositional shift in the hybrid material system. The Ca/P atomic ratios of 25%MK, 50%MK, 75%MK and 100%MK of OD and CP samples are shown in Table 2.

**Table 2:** Ca/P atomic ratios of samples

Samples	Ca/P for samples of OD	Ca/P for samples of CP
25%MK	1.71	1.69
50%MK	1.65	1.61
75%MK	1.52	1.47
100%NK	1.41	1.37

### 3.4 BET

The crystallite size of (HA/MK) composites was estimated *via* the Scherrer equation based on the (211) diffraction peak centered at approximately 2*θ* ≈ 31.8°, which is characteristic of the HA phase. As the MK content increased from 25% to 100%, the calculated crystallite size showed a progressive reduction, decreasing from approximately 37.3 nm in the 25%MK sample to around 21.1 nm in the 100%MK composite. This trend suggested that the incorporation of MK, an amorphous aluminosilicate phase, inhibited the long-range ordering of the HA crystal lattice and disrupted crystal growth, resulting in smaller nanocrystalline domains.^30–34^ The disruption was likely due to the physical presence of MK particles interfering with HA nucleation and the chemical complexity introduced by silicate and aluminate species during synthesis.

This decrease in crystallite size was accompanied by a concurrent reduction in BET surface area, total pore volume, and mean pore diameter, particularly in the (CP) samples (Fig. S1–S3 and Table 3). While smaller crystallites typically led to higher surface areas, the inverse trend observed in this study may be explained by two main factors. First, the nanoscale HA particles may partially occupy the pores of the metakaolin matrix, thereby reducing the accessible pore space available for nitrogen adsorption. Second, and more importantly, the cold pressing process significantly modifies the pore structure of the composites.

**Table 3:** BET analysis of samples

Samples	Surface area (m^2^ g^−1^)	Total pore volume (cm^3^ g^−1^)	Mean pore diameter (nm)
MK	7.94	0.053	26.88
HA	75.32	0.602	31.96
25%MK-OD	67.54	0.492	30.93
25%MK-CP	62.37	0.281	24.88
50%MK-OD	64.32	0.432	29.08
50%MK-CP	57.42	0.253	24.19
75%MK-OD	56.13	0.335	27.57
75%MK-CP	44.23	0.224	23.62
100%MK-OD	53.11	0.281	24.54
100%MK-CP	41.35	0.219	22.04

During cold pressing, the applied pressure brings particles into closer contact, increasing particle packing and deforming contact points between grains. This process promotes densification and can partially collapse or occlude interconnected pores that would otherwise be accessible during nitrogen adsorption measurements. As a result, a portion of the internal porosity becomes inaccessible to nitrogen molecules during BET analysis, leading to a reduction in the measured surface area despite the smaller crystallite size.^27,50^ The BET surface area decreased from approximately 61 m^2^ g^−1^ in 25%MK-CP to 42 m^2^ g^−1^ in the 100%MK-CP samples. Similarly, total pore volume declined from 0.28 cm^3^ g^−1^ to 0.22 cm^3^ g^−1^, and mean pore diameter decreased from 25 nm to about 22 nm over the same composition range. These results indicated that the densification, achieved through cold pressing-along with the inherent packing behavior of fine MK particles, effectively reduced the number and size of accessible pores, thereby limiting the available surface area for nitrogen adsorption during BET analysis.^51^ Therefore, although the crystallite size decreased with increasing MK content, the overall accessible surface area was reduced due to pore occlusion and structural densification. This densification likely contributed to the enhanced mechanical performance observed in parallel to results.

### 3.5 Mechanical and physical properties of the composites

The mechanical and physical characterizations were conducted only on the CP samples, as the OD samples were prepared solely to compare the effect of the cold-pressing conditions on HA integrity.

#### 3.5.1 Compressive strength, DTS, Young modulus and hardness

The mechanical characterization of the cold-pressed samples, as summarized in Table 4, revealed a progressive enhancement in key mechanical parameters-namely, compressive strength, diametral tensile strength (DTS), Vickers hardness, and Young's modulus-with increasing MK content. The compressive strength increased markedly from approximately 10.4 MPa for 25% MK to 16.4 MPa for 100% MK. This enhancement was attributed to the increased reaction leading to zeolitic phases facilitated by the higher MK content, leading to a denser and more cohesive microstructure. The compressive strength results fell within the range of cancellous bone, which is typically 2–20 MPa, suggesting that the material can provide adequate mechanical support for bone scaffold applications.^52^

**Table 4:** Mechanical properties of HA/MK composites (CP)

Tests	25%MK	50%MK	75%MK	100%MK	*p*-Value
Compressive strength (MPa)	10.4 ± 2.7	13.7 ± 1.6	14.9 ± 1.6	16.4 ± 1.3	0.016
DTS (MPa)	6.1 ± 1.2	7.5 ± 1.7	10.3 ± 1.7	11.8 ± 1.8	0.034
Hardness (Hv)	235 ± 25	235 ± 22	214 ± 22	204 ± 36	0.609
Young's modulus (GPa)	5.0 ± 0.29	5.0 ± 0.16	5.2 ± 0.14	5.6 ± 0.20	0.050

Similarly, DTS values demonstrated a positive correlation with MK content, increasing from 6.1 MPa to 11.8 MPa. The rise in tensile strength suggested that the MK matrix formed at higher ratios exhibited superior internal bonding and resistance to tensile stress, reinforcing the mechanical integrity of the composites. This improvement in tensile properties is beneficial for bone scaffold applications, as it helps the scaffold withstand physiological stresses and maintain structural stability during bone regeneration. This result is consistent with that of Ibrahim *et al.*, who studied the effect of metakaolin alkaline activation on HA scaffolds.^53^

The Vickers hardness values remained relatively stable across all formulations, ranging between 204 and 235 Hv. The minimal variation implied that while the bulk matrix densification increased with MK content, surface hardness was less sensitive to MK ratio under Vickers indentation. This may have resulted from the localized nature of the indentation test and possible surface heterogeneities or residual porosity. The hardness value observed in this study was higher than those reported previously.^54^ Hardness values referred to surface resistance to deformation, which is beneficial for maintaining the structural integrity of bone scaffolds during implantation and service in physiological environments.

Young's modulus, representing the stiffness of the composite, also followed a similar upward trajectory. The Young's modulus values were 5.0, 5.0, 5.2, and 5.6 GPa for the samples containing 25%, 50%, 75%, and 100% MK, respectively, indicating a progressive improvement in elastic behavior with increasing MK content. The increase in modulus reflected improved load transfer across the composite and enhanced rigidity, likely resulting from the integration of amorphous metakaolin, which acted as a stiffening agent. The Young's modulus of cancellous bone ranges from 1.0 to 22.3 GPa.^55^

Lastly, ANOVA results indicated that the compressive strength, DTS and Young's modulus are significantly increased with increasing metakaolin content (*p* ≤ 0.05), between 25%MK and 100%MK. Meanwhile, hardness was not statistically significant (*p* ≥ 0.05).

#### 3.5.2 Porosity and density

**Fig. 7:**
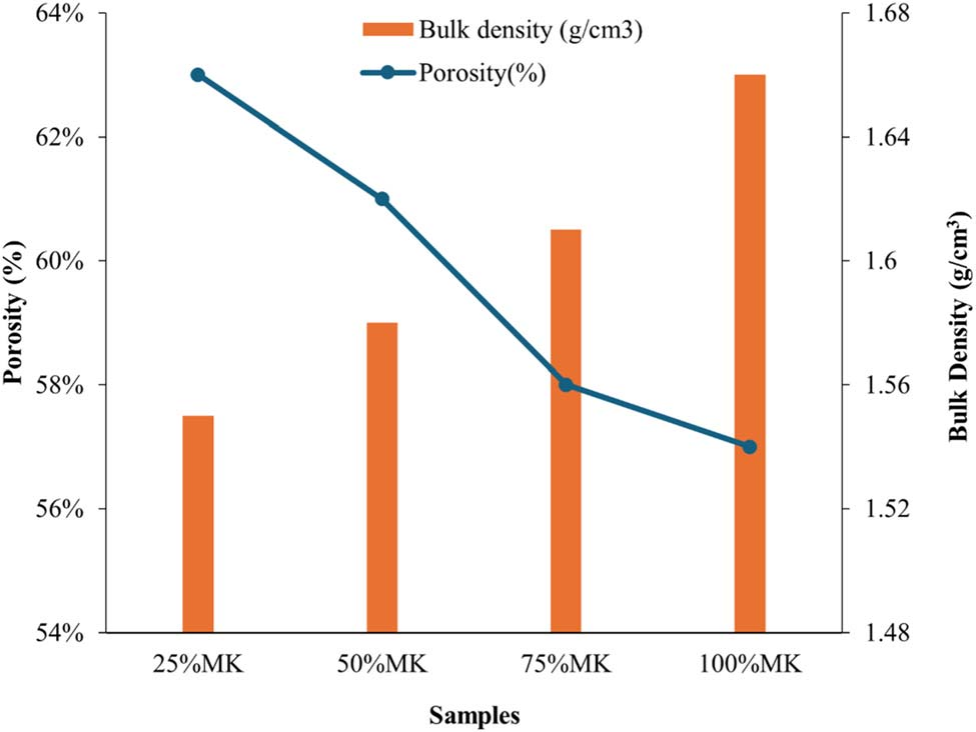
Porosity and bulk density of HA/MK composites (CP).

The Fig. 7 illustrated the variation in porosity (%) and bulk density (g cm^−3^) of cold-pressed composites. The results revealed a clear inverse relationship between porosity and bulk density as MK content increased. At 25% MK, the composite exhibited the highest porosity (63%) and the lowest bulk density (1.57 g cm^−3^), indicating a more open microstructure likely due to inefficient particle packing and higher void content. This was expected in compositions with a lower proportion of the fine, amorphous MK phase, which contributed less effectively to matrix densification.

As the MK content increased to 50% and 75%, porosity declined gradually to approximately 61% and 58%, respectively, while the bulk density increased to 1.59 and 1.61 g cm^−3^. This improvement can be attributed to the filler effect of metakaolin, whose finer particles can occupy interstitial spaces within the HA matrix, thus enhancing packing efficiency and reducing voids. At 100% MK, the composite reached the lowest porosity (55%) and highest bulk density (1.66 g cm^−3^), demonstrating the dominant densifying effect of MK when used as the primary solid phase in the composite.

The increasing bulk density with concurrent reduction in porosity indicates more effective compaction and microstructural consolidation with higher MK content under cold pressing. These structural changes were highly favorable for improving the composite's mechanical properties, as lower porosity typically correlated with greater compressive strength and stiffness. It has been reported that the desired porosity range for cortical bone is 50–80%.^56^

#### 3.5.3 Biodegradability

**Fig. 8:**
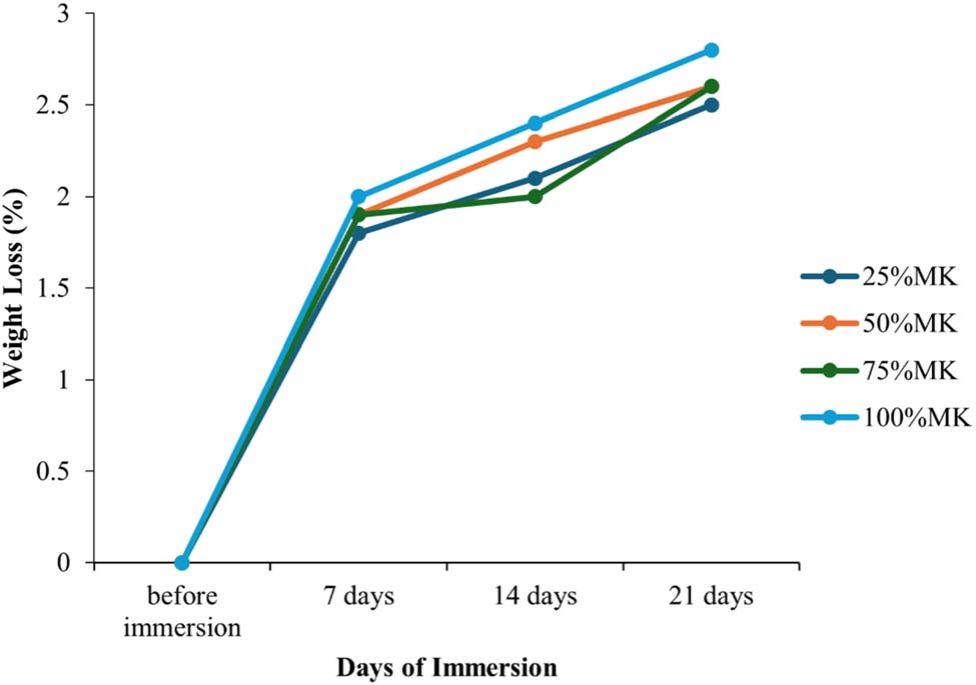
Weight loss (%) of HA/MK composites (CP) immersed in PBS.

The biodegradability of the composite samples immersed in (PBS) was evaluated through weight loss measurements and pH variation analysis over a period of 21 days. As illustrated in Fig. 8, all samples exhibited a gradual increase in weight loss with immersion time, indicating progressive degradation. The 25%MK and 50%MK samples showed moderate degradation, reaching approximately 2.5% and 2.6% weight loss, respectively, after 21 days. The 75%MK sample exhibited slightly lower degradation, possibly due to improved matrix integrity with higher MK content. Interestingly, the 100%MK sample demonstrated the highest weight loss (approaching 2.8%), suggesting that despite the densification observed in previous physical property tests, the absence of HA or CES may have reduced the composite's resistance to PBS-mediated degradation. In parallel, the pH variation of the PBS medium was monitored to assess ion release and dissolution behavior. Fig. S4 reveals a consistent increase in pH for all samples, with the 100%MK specimen showing the highest pH shift from 7.4 to 8.2 over the 21-day period. This alkaline shift can be attributed to the leaching of alkali ions from the geopolymeric network and partial dissolution of residual calcium species from CES or HA phases. The 25%MK and 50%MK samples exhibited a more moderate pH increase, reaching 7.9 and 8.1, respectively, which correlated with their relatively lower weight loss rates. Overall, the results suggested that higher MK content enhanced the rate of biodegradation in PBS, as evidenced by the increasing weight loss and elevated pH, likely due to the higher availability of reactive aluminosilicate species. The observed behavior also aligned with the porosity trends reported earlier, where higher porosity facilitates ion exchange and fluid infiltration, accelerating degradation. These findings are significant for tailoring the degradation rate of bioceramic scaffolds for tissue engineering applications, where gradual resorption is desirable to match tissue regeneration rates.

### 3.6 Biocompatibility and biofunctionality

Based on the results obtained from characterization analyses, mechanical testing, and physical evaluations, the 25% MK sample was selected for further biological and bioactivity assessments to ensure the structural and compositional integrity of HA. This decision was made because samples with 50% MK or higher showed a reduction in porosity and a compromised HA integrity, likely due to ion incorporation or changes in crystallinity.

#### 3.6.1 Bioactivity

**Fig. 9:**
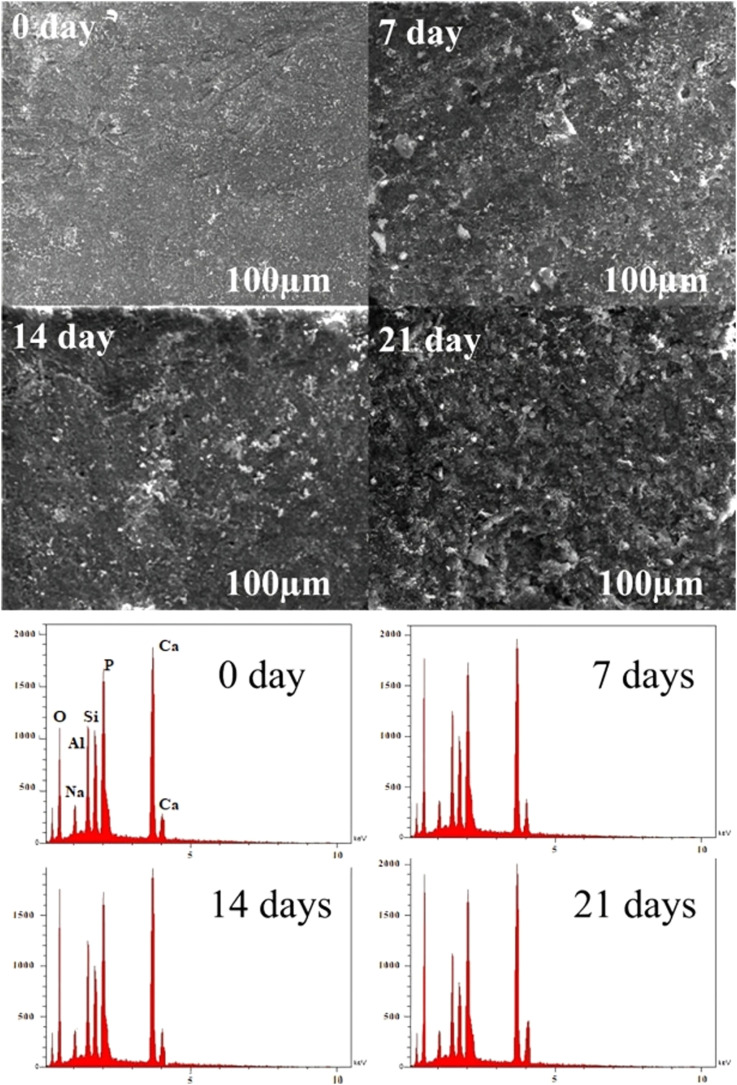
SEM and EDX images of HA/MK (CP) composites after immersion in SBF for 0, 7, 14, and 21 days.

The bioactivity of the scaffold was evaluated by immersing it in SBF for different time periods, and the changes in surface morphology and elemental composition were examined using SEM and EDX analyses (Fig. 9). At 0 days, the scaffold surface appeared relatively smooth and dense with only minor irregularities, and no evidence of apatite deposition was observed, indicating the absence of nucleation activity at this stage. After 7 days of immersion, small granular precipitates began to appear on the surface, suggesting the initiation of apatite nucleation through ion exchange between the scaffold and the SBF. By 14 days, the surface was more uniformly covered with rougher and more porous apatite-like deposits, reflecting significant crystal growth and partial coalescence of calcium-phosphate clusters into a continuous layer.^18,57^ At 21 days, the scaffold surface was completely covered by a dense and compact apatite layer, indicating advanced maturation of the precipitated phase.^18^ EDX analysis further confirmed these observations. Initially, the spectra at 0 days displayed prominent peaks of Ca, P, O, Na, Si, and Al, consistent with the original scaffold composition and showing a Ca/P ratio near to stoichiometric hydroxyapatite (1.69). After 7 days, Ca and P peak intensities increased recorded 1.74 Ca/P molar ratio, while Si and Al peaks diminished, indicating partial surface coverage by bone-like apatite deposits. This trend continued at 14 and 21 days, with Ca and P peaks becoming more dominant and the Ca/P ratio approaching 1.75 and 1.78, It has been reported that the Ca/P molar ratio of biological (bone-like) apatite is either lower than or close to that of stoichiometric HA.^57,58^ Alongside a further increase of O and reduction in Na, Al and Si, confirming that the surface was fully coated with an apatite layer. The progressive development of this Ca-rich apatite surface demonstrated that the scaffold possessed excellent bioactivity.

#### 3.6.2 Antimicrobial assessment

**Fig. 10:**
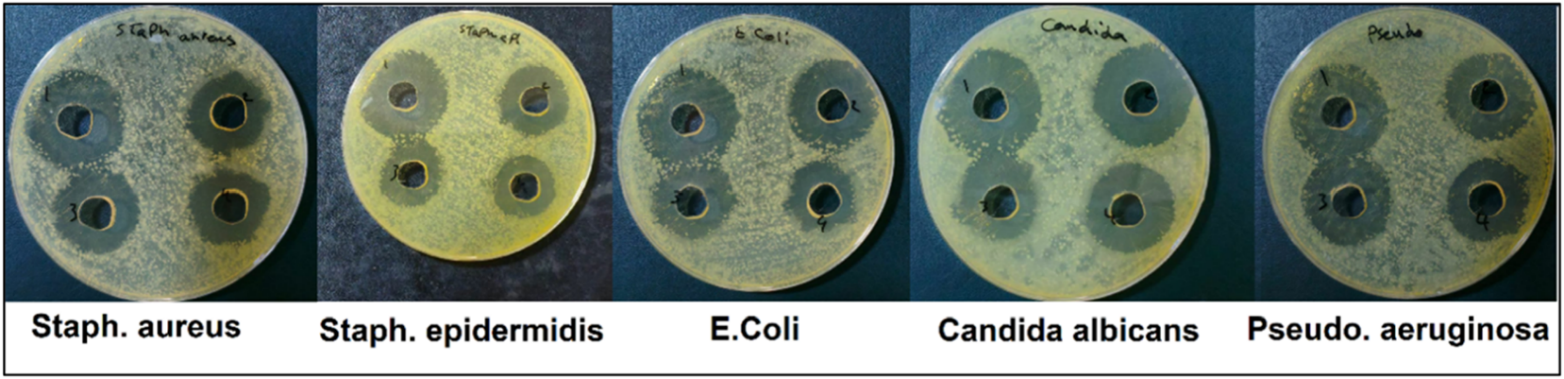
Antimicrobial inhibition zones of the 25%MK scaffold against *Candida albicans*, *Staphylococcus epidermidis*, *Staphylococcus aureus*, *Escherichia coli*, and *Pseudomonas aeruginosa*.

The antimicrobial efficacy of the tested samples was assessed against five microbial strains, including both Gram-positive (*Staphylococcus aureus*, *Staphylococcus epidermidis*) and Gram-negative (*Escherichia coli*, *Pseudomonas aeruginosa*) bacteria, as well as a fungal strain (*Candida albicans*), using the agar well diffusion method at four concentrations (1000, 500, 250, and 125 µg mL^−1^) (Fig. S5). The data revealed a concentration-dependent inhibition pattern, as the zone of inhibition (ZOI) generally decreased with decreasing concentration (Fig. 10 and Table 5). At the highest concentration (1000 µg mL^−1^), the most pronounced antimicrobial activity was observed against *Escherichia coli* and *Staphylococcus epidermidis*, with ZOIs of 29 mm in both cases, indicating strong bactericidal effects. *Staphylococcus aureus* and *Candida albicans* also showed notable inhibition zones of 27 mm each, while *Pseudomonas aeruginosa* exhibited a slightly lower ZOI of 26 mm, suggesting relatively higher resistance among the tested isolates. As the concentration decreased, a gradual decline in antimicrobial activity was observed. At 125 µg mL^−1^, the ZOI values ranged from 22 to 23 mm for all strains, indicating that although the efficacy diminished, the material retained a measurable antimicrobial effect even at the lowest tested dose. This broad activity may be attributed to several factors. First, the bioactive phases or metal ions (*e.g.*, Ca^2+^, Na^+^ and Si^4+^) are known to damage microbial DNA and inhibit replication.^59–61^ Second, nano-HA has been reported to demonstrate antimicrobial activities against Gram-negative and Gram-positive bacteria and fungi.^62^ The sustained activity against *Candida albicans* also supported its potential use in antifungal applications. These results are compatible with several studies on the effect of MK as a strengthening agent for HA.^18^

**Table 5:** Inhibition zones (mm) of 25%MK sample at different concentrations

Isolates	1000 µg mL^−1^	500 µg mL^−1^	250 µg mL^−1^	125 µg mL^−1^
*Candida albicans*	27	26	24	23
*Staphylococcus epidermidis*	29	25	23	22
*Staphylococcus aureus*	27	23	23	22
*Escherichia coli*	29	26	24	23
*Pseudomonas aeruginosa*	26	25	23	23

#### 3.6.3 MTT assay

**Fig. 11:**
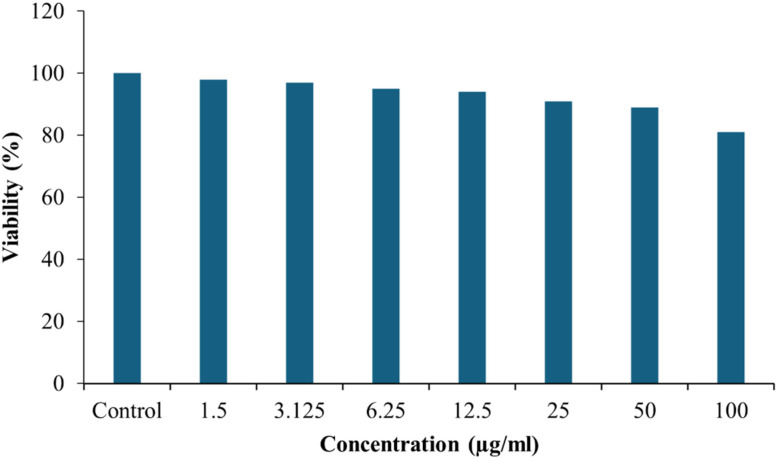
MTT assay results of 25%MK at different concentrations after 48 h incubation with MC3T3-E1 cells.

The MTT assay results conducted on MC3T3-E1 pre-osteoblast cells after 48 hours of incubation demonstrated a concentration-dependent cytotoxic response to the tested material (Fig. 11 and S5). At lower concentrations ranging from 1.5 to 12.5 µg mL^−1^, cell viability remained above 95%, indicating minimal or negligible cytotoxicity and suggesting that the material is biocompatible at these levels. A moderate decline in viability was observed at concentrations of 25 and 50 µg mL^−1^, where the percentage decreased to approximately 90% and 85%, respectively. This slight reduction suggested a mild cytotoxic effect, which may still fall within acceptable biocompatibility limits for biomedical applications. However, at higher concentrations of 100 µg mL^−1^, a significant decrease in cell viability was recorded, with values dropping to around 75% and 60%, respectively. These findings indicated that, while the material exhibited good cytocompatibility at low and moderate doses, elevated concentrations may have induced cytotoxic effects on pre-osteoblast cells. It is important to note that in practical bone scaffold applications, the local concentration of released species is typically much lower and occurs gradually as the scaffold degrades in physiological environments. Therefore, the observed cytotoxicity at high concentrations may represent a worst-case *in vitro* exposure scenario rather than actual physiological conditions. Overall, the results indicate that the 25% MK composite demonstrates good cytocompatibility within biologically relevant concentration ranges and remains a promising candidate for bone tissue engineering applications.

The overall performance of the HA/MK composites was governed by the interaction between HA nanocrystals and the amorphous aluminosilicate phase of metakaolin. Structural analyses (XRD and FTIR) revealed that increasing MK content reduced HA crystallinity and crystallite size, while SEM observations confirmed the formation of a hybrid microstructure where rod-like HA nanoparticles were distributed over plate-like MK particles. Cold pressing enhanced particle packing and reduced pore size, which led to decreased porosity and increased bulk density. These microstructural changes improved load transfer within the composite, resulting in higher compressive strength, tensile strength, and Young's modulus. Such mechanical properties fall within the range of cancellous bone, indicating that the scaffold can provide sufficient structural support during bone regeneration. At the same time, the presence of nanoscale HA and interconnected porosity supports biological performance by promoting apatite formation in simulated body fluid, enabling cell attachment and proliferation, and facilitating nutrient transport. The observed antimicrobial activity further enhances the scaffold's functionality by reducing the risk of post-implantation infection. Therefore, the balance between MK-induced structural densification and HA bioactivity provides a combination of mechanical stability, controlled biodegradation, and biological functionality, which are essential characteristics for bone tissue engineering scaffolds.

## 4. Conclusions

This study successfully developed a sustainable, zero-waste wet chemical precipitation method to synthesize nano-hydroxyapatite from eggshell biowaste and reinforced it with calcined metakaolin for potential biomedical applications. The elimination of conventional washing and filtration steps significantly enhanced material yield and process efficiency, as these steps can lead to partial loss of nanoparticles due to their nanoscale dimensions and high colloidal stability. Structural analyses (XRD, FTIR, SEM-EDX) confirmed the formation of HA with rod-like nanocrystals and effective incorporation of MK, which resulted in reduced crystallinity and crystallite size due to aluminosilicate interactions. Cold pressing improved bulk density, reduced porosity, and enhanced mechanical properties, with compressive strength (16.4 MPa) and diametral tensile strength (11.8 MPa) values within the cancellous bone range. Bioactivity tests demonstrated progressive apatite layer formation on the composite surface in SBF, while antimicrobial evaluations revealed broad-spectrum activity against Gram-positive, Gram-negative, and fungal strains. Cytotoxicity assays indicated excellent biocompatibility, with cell viability exceeding 95% at concentrations up to 12.5 µg mL^−1^. Overall, the developed HA/MK composite exhibits a unique combination of mechanical strength, bioactivity, antimicrobial functionality, and cytocompatibility, supporting its potential as a promising candidate for bone tissue engineering scaffolds and other biomedical applications. Future studies should focus on *in vivo* performance and optimization of degradation rates to further validate its clinical applicability.

## Conflicts of interest

There are no conflict to declare.

## Supplementary Material

RA-016-D6RA01169A-s001

## Data Availability

The data supporting the findings of this study are included within the manuscript and the supplementary information (SI). Supplementary information is available. See DOI: https://doi.org/10.1039/d6ra01169a.

## References

[cit1] Hincke M. T., Nys Y., Gautron J., Mann K., Rodriguez-Navarro A. B., McKee M. D. (2012). Front. Biosci..

[cit2] Daculsi G., Passuti N., Martin S., Deudon C., Legeros R., Raher S. (1990). J. Biomed. Mater. Res..

[cit3] Rivera E. M., Araiza M., Brostow W., Castano V. M., Dıaz-Estrada J., Hernández R., Rodrıguez J. R. (1999). Mater. Lett..

[cit4] FAO , World Food and Agriculture-Statistical Yearbook 2023, 2023

[cit5] Mignardi S., Archilletti L., Medeghini L., De Vito C. (2020). Sci. Rep..

[cit6] Gomez-Vazquez O. M., Correa-Piña B. A., Zubieta-Otero L. F., Castillo-Paz A. M., Londoño-Restrepo S. M., Rodriguez-García M. E. (2021). Ceram. Int..

[cit7] Kamalanathan P., Ramesh S., Bang L. T., Niakan A., Tan C. Y., Purbolaksono J., Chandran H., Teng W. D. (2014). Ceram. Int..

[cit8] Ummartyotin S., Tangnorawich B. (2015). Colloid Polym. Sci..

[cit9] Muthu D., Kumar G. S., Kattimani V., Viswabaskaran V., Girija E. (2020). Ceram. Int..

[cit10] Agbeboh N., Oladele I., Daramola O., Akinwekomi A., Tanimola M., Olasukanmi O. (2022). Futa J. Eng. Eng. Technol.

[cit11] Adamski R., Siuta D. (2021). Molecules.

[cit12] Pádua A. S., Figueiredo L., Silva J. C., Borges J. P. (2023). Prog. Biomater..

[cit13] Rezania N., Asadi-Eydivand M., Abolfathi N., Bonakdar S., Mehrjoo M., Solati-Hashjin M. (2022). J. Mater. Sci.: Mater. Med..

[cit14] Pérez E. (2021). J. Mater. Sci..

[cit15] Ko H.-S., Lee S., Lee D., Jho J. Y. (2021). Nanomaterials.

[cit16] Satish P., Hadagalli K., Praveen L. L., Nowl M. S., Seikh A. H., Alnaser I. A., Abdo H. S., Mandal S. (2023). Inorganics.

[cit17] Obada D., Dauda E. T., Abifarin J., Bansod N. D., Dodoo Arhin D. (2020). Mater. Today: Proc..

[cit18] Obada D. O., Osseni S. A., Sina H., Salami K. A., Oyedeji A. N., Dodoo-Arhin D., Bansod N. D., Csaki S., Atta A. Y., Fasanya O. O., Sowunmi A. R. (2021). Appl. Clay Sci..

[cit19] Kokubo T., Takadama H. (2006). Biomaterials.

[cit20] Khandelwal H., Prakash S. (2016). J. Miner. Mater. Char. Eng..

[cit21] Agbabiaka O., Oladele I., Akinwekomi A., Adediran A., Balogun A. O., Olasunkanm O., Olayanju T. (2020). Sci. Afr..

[cit22] Pu'ad N. M., Alipal J., Abdullah H., Idris M., Lee T. (2021). Mater. Today: Proc..

[cit23] Tchakouté H. K., Melele S. J., Djamen A. T., Kaze C. R., Kamseu E., Nanseu C. N., Leonelli C., Rüscher C. H. (2020). Appl. Clay Sci..

[cit24] Khaled Z., Mohsen A., Soltan A., Kohail M. (2023). Ain Shams Eng. J..

[cit25] Davidovits J., Ceram J. (2017). Sci. Technol..

[cit26] Sudagar A. J., Andrejkovičová S., Rocha F., Patinha C., Soares M. R., Velosa A. L., Silva E. F. d. (2021). Minerals.

[cit27] Li L., Yang F., Liu C., Chen Z., Ge J., Hou Z. (2025). Ceram. Int..

[cit28] Reger N. C., Bhargava A. K., Ratha I., Kundu B., Balla V. K. (2019). Ceram. Int..

[cit29] Baskaran T., Mohammad N. F., Md Saleh S. S., Mohd Nasir N. F., Mohd Daud F. D. (2021). Synthesis methods of doped hydroxyapatite: a brief review. *J. Phys.: Conf. Ser.*.

[cit30] Xu J., Khor K. A. (2007). J. Inorg. Biochem..

[cit31] Tavafoghi M., Kinsella J. M., Gamys C. G., Gosselin M., Zhao Y. F. (2018). Ceram. Int..

[cit32] Wang M., Wang L., Shi C., Sun T., Zeng Y., Zhu Y. (2016). Phys. Chem. Chem. Phys..

[cit33] Farkas N.-I., Turdean G. L., Bizo L., Marincaş L., Cadar O., Barbu-Tudoran L., Réka B. (2023). Ceram. Int..

[cit34] Goldberg M., Protsenko P., Smirnov V., Antonova O., Smirnov S., Konovalov A., Vorckachev K., Kudryavtsev E., Barinov S., Komlev V. (2020). J. Mater. Res. Technol..

[cit35] Khalid M., Jikan S. S. B., Adzila S., Murni Z., Badarulzaman N., Rosley R., Hameed M. U. (2022). Biointerface Res. Appl. Chem..

[cit36] Lala S. D., Barua E., Deb P., Deoghare A. B. (2021). Mater. Today Commun..

[cit37] Goh K. W., Wong Y. H., Ramesh S., Chandran H., Krishnasamy S., Sidhu A., Teng W. (2021). Ceram. Int..

[cit38] Onutai S., Osugi T., Sone T. (2023). Materials.

[cit39] Onutai S., Sato J., Osugi T. (2023). J. Solid State Chem..

[cit40] Caciatori R. A., Dal-Bó A. G., Bernardin A. M. (2024). Miner. Eng..

[cit41] Tian K. V., Mahmoud M. Z., Cozza P., Licoccia S., Fang D.-C., Di Tommaso D., Chass G. A., Greaves G. N. (2016). J. Non-Cryst. Solids.

[cit42] Horta M. K. d. S., Moura F. J., Aguilar M. S., Westin C. B., Campos J. B. d., Peripolli S. B., Ramos V. S., Navarro M. I., Archanjo B. S. (2020). Mater. Res..

[cit43] Ibrahim A.-R., Zhou Y., Li X., Chen L., Hong Y., Su Y., Wang H., Li J. (2015). Mater. Res. Bull..

[cit44] Trzaskowska M., Vivcharenko V., Benko A., Franus W., Goryczka T., Barylski A., Palka K., Przekora A. (2024). Sci. Rep..

[cit45] N'Guessan N. E., Joussein E., Courtin-Nomade A., Paineau E., Soubrand M., Grauby O., Robin V., Cristina C. D., Vantelon D., Launois P. (2021). Appl. Clay Sci..

[cit46] Tan C. Y., Singh R., Teh Y. C., Tan Y. M., Yap B. K. (2015). Int. J. Appl. Ceram. Technol..

[cit47] Aminian A., Solati-Hashjin M., Samadikuchaksaraei A., Bakhshi F., Gorjipour F., Farzadi A., Moztarzadeh F., Schmücker M. (2011). Ceram. Int..

[cit48] Wakamura M., Kandori K., Ishikawa T. (2000). Colloids Surf., A.

[cit49] Nie Y., Hu C., Kong C. (2012). J. Hazard. Mater..

[cit50] Grasso S., Biesuz M., Zoli L., Taveri G., Duff A. I., Ke D., Jiang A., Reece M. J. (2020). Adv. Appl. Ceram..

[cit51] Sinha P., Datar A., Jeong C., Deng X., Chung Y. G., Lin L.-C. (2019). J. Phys. Chem. C.

[cit52] Chen P.-Y., McKittrick J. (2011). J. Mech. Behav. Biomed. Mater..

[cit53] Ibrahim W. M. A. W., Abdullah M. M. A. B., Jamil N. H., Mohamad H., Salleh M. A. A. M., Sandu A. V., Vizureanu P., Baltatu M. S., Sukmak P. (2023). Appl. Sci..

[cit54] Obada D., Dauda E., Abifarin J., Bansod N., Dodoo-Arhin D. (2021). Mater. Today: Proc..

[cit55] Wu D., Isaksson P., Ferguson S. J., Persson C. (2018). Acta Biomater..

[cit56] Balasubramani V., Jeganathan R., Kumar S. D. (2023). Mater. Today: Proc..

[cit57] Twinprai N., Sutthi R., Ngaonee P., Chaikool P., Sookto T., Twinprai P., Mutoh Y., Chindaprasirt P., Laonapakul T. (2024). Arab. J. Chem..

[cit58] Eliaz N., Metoki N. (2017). Materials.

[cit59] Cabezas-Pizarro J., Redondo-Solano M., Umaña-Gamboa C., Arias-Echandi M. L. (2018). Rev. Argent. Microbiol..

[cit60] Dizaj S. M., Lotfipour F., Barzegar-Jalali M., Zarrintan M. H., Adibkia K. (2014). Mater. Sci. Eng., C.

[cit61] Bivehed E., Hellman B., Fan Y., Haglöf J., Buratovic S. (2023). Mutat. Res., Genet. Toxicol. Environ. Mutagen..

[cit62] Nurdiana Dewi M. G., Gustiono D., Kurnia D., Cahyanto A. (2025). Eur. J. Dent..

