# Supplementary material for: Eggshell waste derived nano-hydroxyapatite/metakaolin composites for bone scaffold applications
Source: RSC Adv. 2026 Apr 17;16(22):20051–64. doi: 10.1039/d6ra01169a (PMC13090087; doi:10.1039/d6ra01169a)
Supplement: RA-016-D6RA01169A-s001 [file RA-016-D6RA01169A-s001.pdf]

Supplementary Information (SI) for RSC Advances.
This journal is © The Royal Society of Chemistry 2026

Supplementary Information

Eggshell Waste Derived Nano-Hydroxyapatite/Metakaolin Composites for Bone Scaffold Applications

Zaid Kareem[a,b*] and Ersan Eyiler[b,c,d]

[a]Prosthetics and Orthotics Engineering Department, University of Kerbala, Iraq

[b]Advanced Materials and Nanotechnology Department, Cukurova University, Adana, Turkey

[c]Department of Chemical Engineering, Cukurova University, Adana, Turkey

[d]Tissue Engineering Department, Cukurova University, Adana, Turkey

Correspondence to: Zaid Kareem (E-mail: zaltaey88@gmail.com)

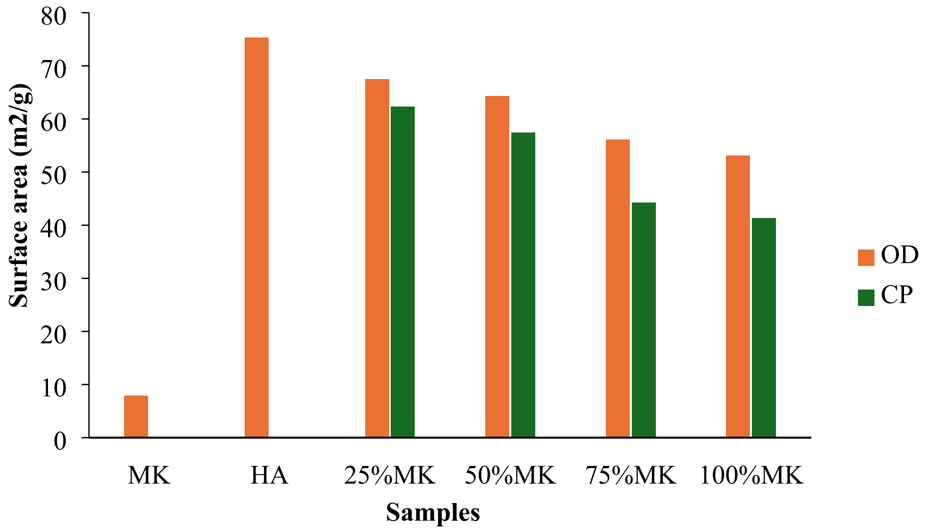

Fig. S1. Surface area of MK, HA and HA/MK composites (CP and OD).

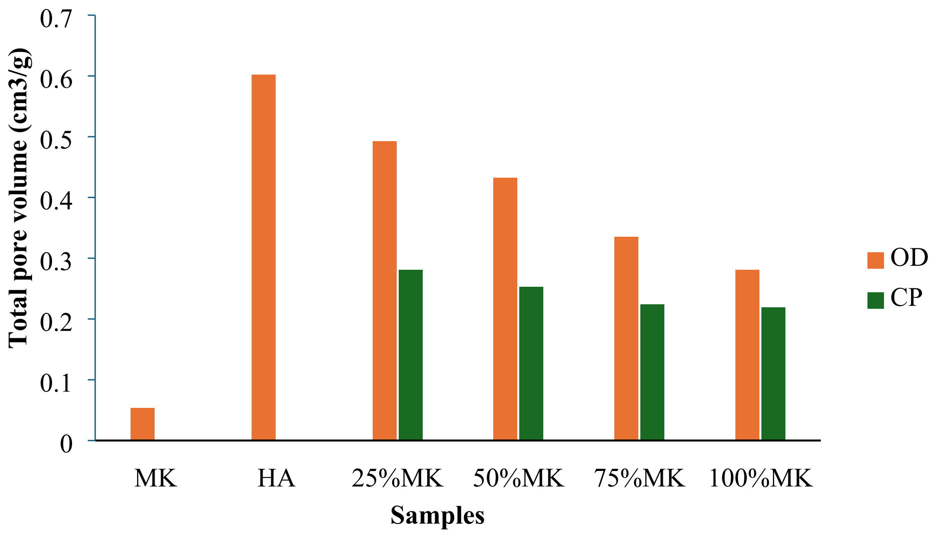

Fig. S2. Total pore volume of MK, HA and HA/MK composites (CP and OD).

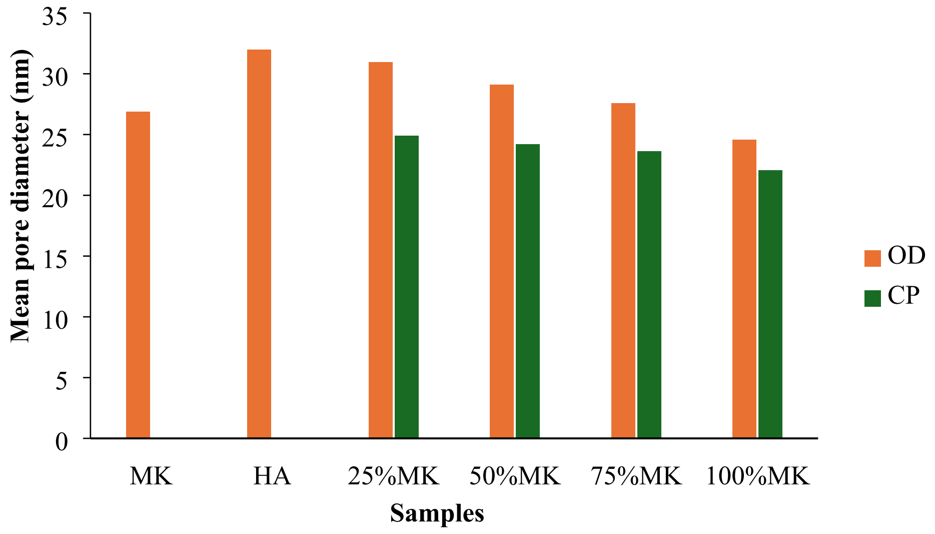

Fig. S3. Mean pore diameter of MK, HA and HA/MK composites (CP and OD).

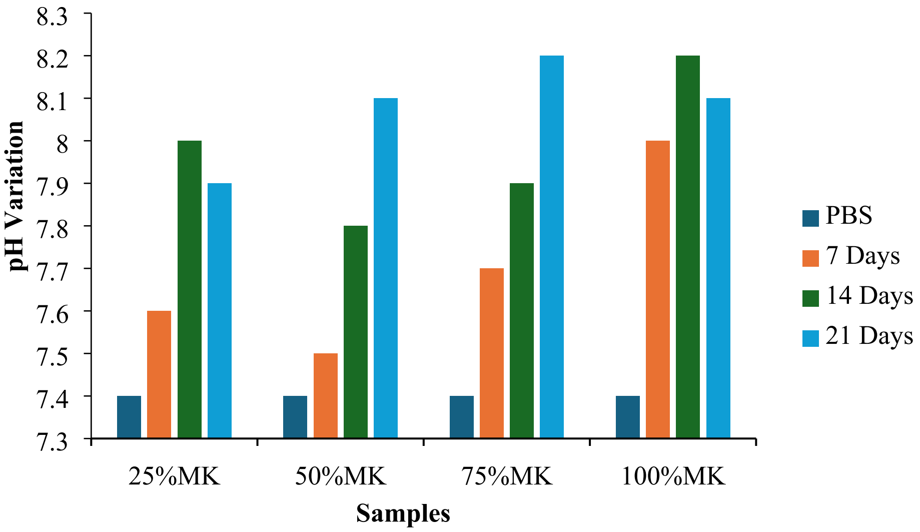

Fig. S4. pH variation of PBS under immersion of HA/MK composites (CP) for 7, 14 and 21 days.

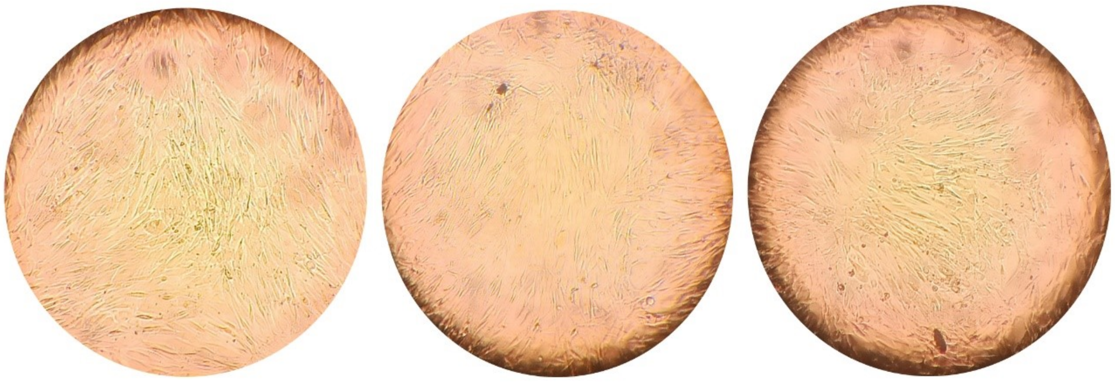

1.5 µg/ml 3.125 µg/ml 6.25 µg/ml

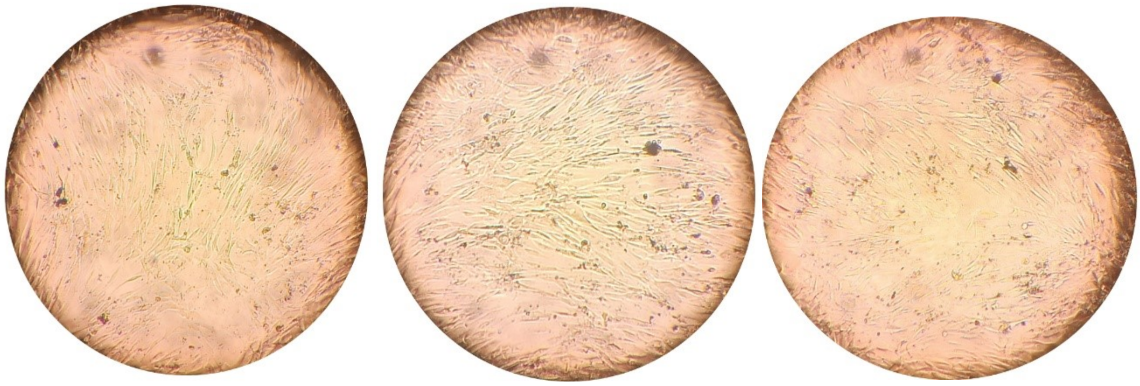

12.5 µg/ml 25 µg/ml 50 µg/ml

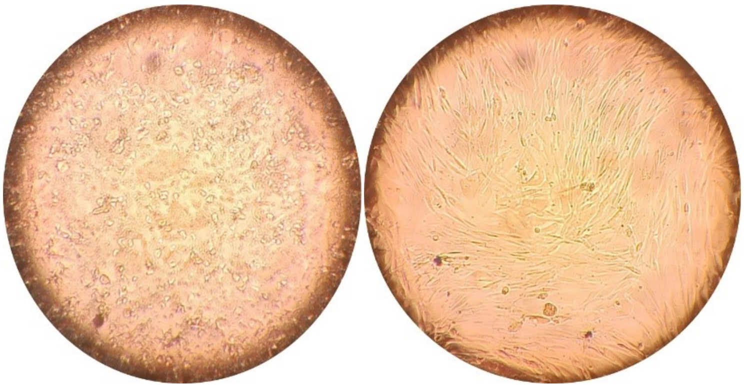

100 μg/ml Control

Fig. S5. Optical microscope images of MC3T3-E1 cells exposed to varying concentrations of 25%MK after 48 h at 37 °C.
